# Structure–Function Relationship and Vision-Related Quality of Life in Glaucoma Secondary to Anterior Uveitis: Comparison with Open Angle Glaucoma

**DOI:** 10.3390/jcm10184231

**Published:** 2021-09-18

**Authors:** Ji-Young Lee, Jin-Ho Kim, Tae-Yoon La, Jin-A Choi

**Affiliations:** 1 Department of Ophthalmology and Visual Science, Daejeon St. Mary’s Hospital, College of Medicine, The Catholic University of Korea, 64 Daeheung-ro, Jung-gu, Daejeon 34943, Korea; lycath1122@gmail.com; 2 Department of Ophthalmology and Visual Science, Seoul St. Mary’s Hospital, College of Medicine, The Catholic University of Korea, 222 Banpo-daero, Seocho-gu, Seoul 06591, Korea; dmtko1@naver.com; 3 Department of Ophthalmology and Visual Science, St. Vincent’s Hospital, College of Medicine, The Catholic University of Korea, 93 Jungbu-daero, Paldal-gu, Suwon 16247, Korea

**Keywords:** structure–function relationship, quality of life, open angle glaucoma, uveitis

## Abstract

Purpose: The aim of this study is to investigate the structure–function characteristics and vision-related quality of life (VR-QoL) in uveitic glaucoma (UG) compared with open-angle glaucoma (OAG). Method: The study included 69 patients with UG and 138 patients with primary open angle glaucoma, normal-tension glaucoma. A 25-item National Eye Institute Visual Function Questionnaire (VFQ-25) was used to evaluate the patients’ VR-QoL. The retinal nerve fiber layer thickness (RNFLT) was measured using optical coherence tomography, and the integrated visual field (IVF) was determined using the best location method. Results: There were no significant differences in the binocular IVF and mean deviation (MD) of the affected eye between the OAG and UG group, whereas the average RNFLT was significant thinner in the OAG group (*p* = 0.008). The patients with UG showed a significantly lower composite score and five subscales of the VFQ-25, compared with those with OAG (*p* < 0.05, for all, general linear model). Multivariate linear regression analyses showed that the composite score showed the strongest associations with the whole IVF (β = 1.240, *p* < 0.001) in the OAG group, whereas in the UG group, a significant association was seen only with the inferior VF of the affected eye (β = 0.596, *p* = 0.038). Conclusions: The eyes with UG exhibited distinctive structure–function characteristics and worse VR-QoL that differentiated them from OAG eyes.

## 1. Introduction

Uveitis, which is the most common inflammatory ocular disorder, involves inflammation of the middle layer of the eye [1,2]. The prevalence of uveitis is highest in the young-to-middle-aged population [2]. When accompanied by glaucoma, uveitis can lead to permanent vision loss [3,4]. Among the various forms of uveitis, anterior uveitis is most commonly complicated by glaucoma [5]. Glaucoma secondary to uveitis is one of the important causes of irreversible sight loss in glaucoma, which is challenging to detect and manage [6,7].

Patients with uveitis suffer from decreased subjective visual function due to low visual acuity (VA) and visual field (VF) damage in the affected eye; this can also lead to depression [8,9]. Glaucoma can potentially worsen the vision-related quality of life (VR-QoL) in uveitis patients, given that glaucoma alone reduces the patient-reported VR-QoL [10,11,12]. In glaucoma patients, greater VF loss and bilateral involvement are associated with a poorer VR-QoL [10]. The function of the healthier eye and binocular integrated visual field (IVF) are important determinants of VR-QoL in glaucoma patients [13,14]. Although the VA in the healthier eye is known to largely determine the VR-QoL in glaucoma patients [15], the VA of the affected eye is a major determinant in those with uveitis [16,17]. Despite the frequent involvement of glaucoma in anterior uveitis, and the potential difference in the impact of VF damage on the VR-QoL between uveitis and glaucoma patients, the VR-QoL has not been well characterized in patients with uveitis complicated by glaucoma.

The pathophysiological mechanisms of secondary glaucoma due to anterior uveitis are likely different from those of glaucoma. The levels of aqueous proteins are elevated in anterior uveitis [6]. Additionally, proinflammatory cytokines, such as interleukin (IL)-6, IL-8, monocyte chemotactic protein-1, and tumor necrosis factor-α, are elevated in uveitic glaucoma (UG) [18,19]. The inflammatory nature of the disease, the high and widely fluctuating intraocular pressure (IOP), and the associated transient hemodynamic instability of the eye can render the optic nerve head more vulnerable to glaucomatous damage. Clinically, UG is often associated with a more aggressive course compared to open angle glaucoma (OAG) [5,20]. A recent study showed that, in patients with glaucoma associated with uveitis, VF loss occurred twice as fast as in glaucoma patients without uveitis [21]. However, the difference of the structure–function relationship between glaucoma due to anterior uveitis and OAG is not sufficiently known.

In this study, we compared the structure and function parameters between patients with anterior uveitis and glaucoma and age-matched patients with OAG. A comprehensive evaluation of the VR-QoL was conducted to determine the influence of various VF parameters on the VR-QoL in patients with UG, and in those with OAG, focusing on the possible differences in these associations.

## 2. Patients and Methods

This study was approved by the Institutional Review Board of the Catholic University of Korea (IRB Number: VC19RESI0098). The need for written informed consent was waived by our Review Board. All procedures adhered to the tenets of the Declaration of Helsinki. In this cross-sectional study, patients with a diagnosis of recurrent hypertensive anterior uveitis with glaucoma (UG) and those diagnosed with open-angle glaucoma (OAG) were enrolled retrospectively from the clinical database of the Glaucoma Clinic of St. Vincent’s Hospital, College of Medicine, The Catholic University of Korea, from April 2016 to May 2018. We adopted a propensity score-matching strategy to compare the two groups and performed one-to-two matching of the UG and OAG group patients based on age, using the greedy method [22]. We considered age because light sensitivities are decreased with aging [23]. In total, 69 patients with UG and 138 age-matched OAG patients were finally included.

The patients’ medical records were examined, and age, gender, best-corrected VA, slit-lamp examination, Goldmann applanation tonometry, gonioscopic examination, dilated fundus examination, stereoscopic optic disc photography, red-free RNFL photography, standard automated perimetry (SAP; performed using the 24-2 SITA program and a Humphrey Visual Field Analyzer; Carl Zeiss Meditec, Dublin, CA, USA), optical coherence tomography (OCT; Cirrus OCT; Carl Zeiss Meditec) data, and visual function questionnaire-25 (VFQ-25) data were included. The duration of follow-up, number of recurrences, and peak IOP were recorded in patients with UG. Demographic information and clinical data were collected at the nearest time to when the questionnaire was conducted. All eligible patients were required to have a normal anterior chamber and open-angle on slit-lamp and gonioscopic examinations. The available peripapillary RNFL scans were analyzed using the same OCT device (Cirrus HD-OCT; Carl Zeiss Meditec). Reliable results of more than two consecutive VF tests (false-positive error rate < 25%, false-negative error rate < 25%, fixation loss < 20%) were included in the analysis. The exclusion criteria were as follows: a history of any retinal disease including diabetic retinopathy; other glaucoma diagnosis, including pigment dispersion syndrome and pseudoexfoliation; optic nerve diseases except for glaucoma; and a history of systemic medication use or a cerebrovascular event that could affect the VF. Patients with poor quality OCT images due to media opacity were also excluded. In the case of OAG patients, we excluded eyes with a history of ocular trauma or surgery, including trabeculectomy or glaucoma drainage device implantation, with the exceptions of uncomplicated cataract surgery.

Glaucoma was defined as the presence of glaucomatous optic neuropathy (thinning of the neuroretinal rim, peripapillary hemorrhage, or localized pallor) in association with a typical, reproducible VF defect on SAP. A glaucomatous VF was defined by a glaucoma hemifield test result outside of the normal limits and the presence of at least three contiguous points in the pattern deviation plot with *p*-values < 5%, at least one of which had a *p*-value < 1%, on two consecutive reliable SAP examinations. The inclusion criteria for UG were a diagnosis of recurrent anterior uveitis with three or more episodes of recurrences, a history of elevated IOP (>21 mmHg) based on Goldmann applanation tonometry (on at least two separate occasions), and a presence of glaucoma [24,25]. We defined OAG as the presence of glaucomatous optic nerve damage and associated visual field defect without ocular disease or conditions that may elevate the IOP. Inclusion criteria for primary open angle glaucoma (POAG) patients required a history of elevated IOP (>21 mmHg) either with or without treatment. Inclusion criterion for normal tension glaucoma (NTG) patients was a history of untreated peak IOP of 21 mmHg or less.

## 3. VF Examination

The IVFs were derived from the right and left VFs for each patient using the best location method described by Nelson-Quigg et al. [26]. A total of 52 threshold values of the 24-2 SITA VF were compared to determine the optimal threshold value between the two eyes. The IVF threshold values were then calculated.

The mean deviation (MD) values for the IVF were calculated using the following formula [27]:

Expected threshold (ET) = measured threshold − total deviation (TD).

The integrated ET values were calculated using the best location method. An integrated TD map was estimated using the following formula: integrated TD = Integrated threshold − ET. The integrated MD values were calculated by summing the integrated TD values, and then dividing this sum by 52 [27]. Then, the integrated TD values were divided into 26 each at the superior and inferior hemifields to obtain the superior and inferior integrated MD values, respectively.

In the OAG group, one of the two affected VF eyes were selected for the study. When both eyes were eligible, the more affected eye was selected for inclusion. In the UG group, the affected eye was included in the UG group. If both eyes were eligible for the UG group (n = 11), the more affected eye with more severe visual field impairment was chosen.

The affected eye TD was evaluated using the decibel (dB) (10 × log (1/Lambert)) scale. To calculate the mean TD of each sector, the dB level in each location of the TD field was converted to a linear scale before averaging the data within each sector. Then, the averaged data were converted back to decibel units. The mean TD of the superior VF was calculated as the mean VF TD value of 26 test points in the superior hemifield, and that of the inferior VF was the mean of 26 test points in the inferior hemifield (excluding blind spots, as described in the previous research) [28].

## 4. Optical Coherence Tomography

All the patients underwent a spectral-domain OCT. An optic disc scan (optic disc cube, 200 × 200 protocol) and a macular scan (macular cube, 512 × 128 protocol) were acquired by the same operator, on the same day, for the RNFLT and ganglion cell-inner plexiform layer thickness (GCIPLT) measurements, respectively. In the patients with UG, the OCT examination was performed during quiescent periods. Only well-focused, well-centered images, without eye movement and with a signal strength ≥ 6/10 were selected. For the optic disc cube scans, we measured the average peripapillary RNFLT from each of the four quadrants in each 12 clock-hour sector, as well as the vertical C/D ratios and cup volume. In the macular cube scan, the average GCIPLT and six sectoral (superotemporal, superior, superonasal, inferonasal, inferior, and inferotemporal) GCIPLTs in an elliptical annulus were recorded [29,30]. The sectors of the peripapillary RNFL were defined as a superior (the average of measurements in clock-hour segments 10, 11, 12, 1, 2, and 3) or an inferior RNFL (the average of measurements in clock-hour segments 5, 6, 7, and 8); the four and nine clock-hour segments were excluded, as previously described [31].

## 5. Visual Function Questionnaire-25 (VFQ-25)

The patient VR-QoL was evaluated using a validated Korean version of the National Eye Institute Visual Functioning Questionnaire-25 (NEI-VFQ-25) [32]. The VFQ-25 comprises 12 subscales and 25 vision-related questions; the average subscale scores are transformed to a 0–100 scale. The subscales are as follows: general health, general vision, ocular pain, near activities, distance activities, vision-specific social functioning, vision-specific mental health, vision-specific role difficulties, vision-specific dependency, driving, color vision, and peripheral vision. A composite score was calculated by averaging the scores of the 11 subscales (i.e., excluding the general health subscale). A detailed description of the NEI-VFQ-25 is provided elsewhere [15,17,33]. The questionnaire was administered by a trained interviewer. The patients were contacted when they visited the clinic for an ophthalmologic examination, and the questionnaires were administered to those that consented.

## 6. Data Analysis

Comparisons of the baseline characteristics between the OAG and UG patients were performed using Student’s *t*-tests for continuous variables, such as demographic characteristics, VF parameters, and peripapillary RNFL/GCIPL parameters. Chi-square analyses were used to compare the groups in terms of categorical variables. In addition, to compare the group differences between OAG and UG of the subscale and composite NEI VFQ-25, potential confounding factors such as age, whole IVF, visual acuity, number of anti-glaucoma medication, VF MD, average RNFLT, and average GCIPT were included in a general linear model.

We analyzed the correlations of the subscale and composite NEI VFQ-25 with the calculated MD values using Spearman’s correlation. To determine the effect of each VF parameter on the composite scores of the OAG and UG groups, univariate and multivariate linear regression analyses were conducted. The dependent variable was the composite NEI VFQ-25 score; and the independent variables were age; binocular whole IVF; superior and inferior IVF; VA; average IOP; number of anti-glaucoma medications; whole VF; superior, inferior, and center VF; average, superior, and inferior RNFLT; and the average GCIPLT of the affected eyes in each group. In multivariate regression analyses of the composite VFQ-25 score, which included the VF parameters, we first adjusted for age (Model one). Next, we adjusted for age, VA, and the number of medications, which showed trends toward significant differences (*p* < 0.200) In the multivariate linear regression analyses, the structural parameters were not adjusted due to their high collinearity with the VF parameters. For statistical analyses, SPSS statistical software for Windows (version 20.0; IBM Corp., Armonk, NY, USA) and XLSTAT-Premium (Addinsoft, New York, NY, USA) were used. A *p*-value < 0.05 was considered statistically significant.

## 7. Results

This study included 69 patients with UG and 138 age- and average peripapillary RNFLT-matched OAG patients. In the OAG group, 59 patients (42.8%) with POAG and 79 patients (57.2%) with NTG were included. In the UG group, the mean follow-up duration was 34.9 ± 39.0 months. The average number of recurrences was 0.6 ± 0.9 per year, and 15 patients (21.7%) had glaucoma surgery during the follow-up period.

There were no significant differences in age and gender between the OAG and UG groups (*p* = 0.314, 0.175, respectively) (Table 1). Regarding the ocular characteristics of the affected eyes, the UG group showed a significantly worse VA (*p* < 0.001), a higher average IOP (*p* < 0.001) and a higher peak IOP (*p* < 0.001) compared to the OAG group (Table 1).

**Figure 1:**
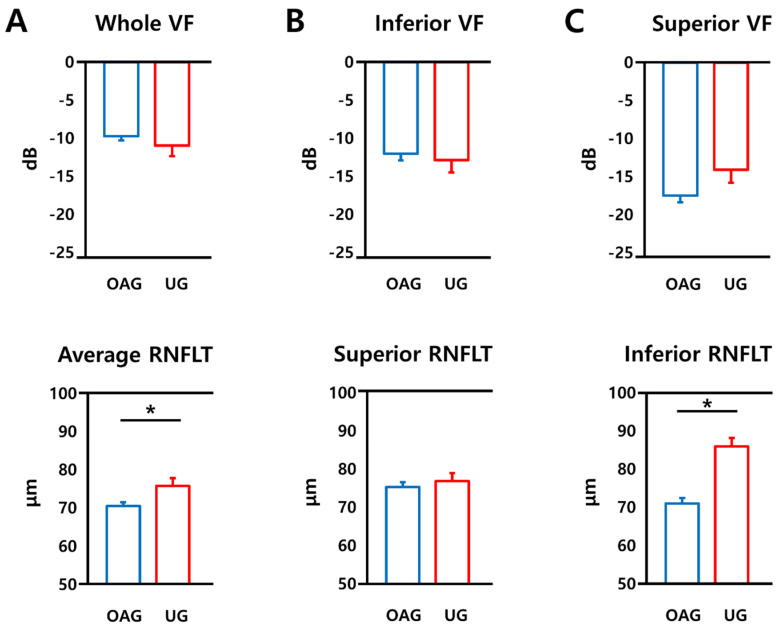
Comparisons of functional and structural parameters between OAG and UG eyes. The mean deviation (MD) of the whole (**A**), inferior (**B**), superior (**C**) visual field and the corresponding average (**A**), superior (**B**), inferior (**C**) and retinal nerve fiber layer thickness are compared between the uveitic glaucoma (UG) group and the open angle glaucoma (OAG) group. * Significant *p*-values < 0.05.

The VF characteristics, the regional VF threshold values, and the corresponding structural parameters of the OAG and UG groups are shown in Table 1 and Figure 1. The binocular IVF of the whole, superior, and inferior fields did not differ between the groups (*p* = 0.341, 0.364, and 0.381, respectively). There were no significant differences in the MD values of the affected eye between the OAG and UG groups (*p* = 0.310). In the comparison of the structural parameters, there were no significant differences of the superior RNFLT and the average GCIPLT, while the average RNFLT and the inferior RNFLT were significantly thinner in OAG (*p* = 0.008, <0.001, respectively; Student’s *t*-test, Table 1 and Figure 1).

Regarding the VFQ-25 scores, the UG group had a significantly lower composite score and five subscales (distance vision, vision-specific social function, vision-specific role difficulties, color vision, and peripheral vision) after adjusting for potential confounding factors (*p* < 0.05 for all, general linear model, Table 2).

**Figure 2:**
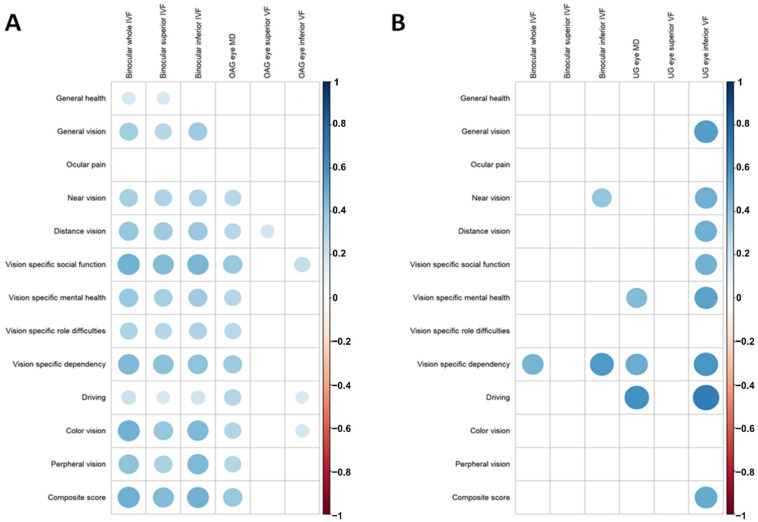
Correlation coefficients between vision-related quality of life and entire/sectoral VF in OAG (**A**) and UG (**B**). OAG, open angle glaucoma; UG, uveitic glaucoma; VF, visual field; MD, mean deviation. Correlation coefficients with statistical significance (*p* < 0.05) are shown. Color and size of the circle provide the magnitude of correlation.

In the patients with OAG, the binocular whole IVF had the strongest correlation with the composite score (r = 0.487, *p* < 0.001), followed by the binocular inferior IVF (r = 0.478, *p* < 0.001) and the binocular superior IVF (r = 0.431, *p* < 0.001) (Table 3). The whole VF of the affected eye had a modest correlation with the composite score (r = 0.372, *p* < 0.001) and nine subscale scores (Figure 2A). However, in the UG group, none of the binocular VF parameters had a significant correlation with the composite score (Figure 2B). The inferior VF of the affected eye showed the highest correlation with the composite score (r = 0.509, *p* = 0.006) and seven subscale scores.

In the univariate linear regression analyses, age, the binocular IVF (whole, superior and inferior IVF), the whole VF, and the superior and inferior VF significantly affected the composite VFQ-25 scores in the OAG group (Table 3). In the UG group, the number of anti-glaucoma medications, the inferior VF, and the average and superior RNFLT significantly affected the composite score. In the multivariate analyses, the binocular inferior IVF (adjusted R^2^ = 0.221, β = 1.259, confidence interval (CI): 0.831–1.688) and the binocular whole IVF (R^2^ = 0.226, β = 1.240, CI: 0.825–1.655) showed the strongest associations with the composite score, after adjusting for age, VA, and the number of anti-glaucoma medications in the OAG group (Table 4). This was followed by the binocular superior IVF (R^2^ = 0.176, β = 0.898, CI: 0.541–1.254) and the whole VF of the affected eye (R^2^ = 0.125, β = 0.641, CI: 0.321–0.962). In the UG group, the inferior VF of the affected eye had a significant correlation with the composite score (R^2^ = 0.236, β = 0.596, CI: 0.037–1.156).

## 8. Discussion

In the present study, we investigated the structure–function relationship and subjective visual function in patients with glaucoma secondary to recurrent anterior uveitis and OAG. The general linear model was applied to adjust for potential confounding factors such as the structural and functional metrics of glaucomatous damage, age, visual acuity, and the number of anti-glaucoma medications. We found that the patients with UG were associated with lower composite VFQ-25 scores and five subscales (distance vision, vision-specific role difficulties, vision-specific social function, color vision, and peripheral vision), compared to the OAG patients (Table 2). Previous studies [9,17,34] have suggested that uveitis tends to accompany a lower VR-QoL compared to normal subjects. However, to our knowledge, this is the first study to compare the VR-QoL between OAG and UG patients.

In a comparison of clinical characteristics, the UG eyes had a significantly lower VA and a higher IOP compared to those with OAG (Table 1). Poor vision is associated with poor VR-QoL in uveitis [17]. A high frequency of recurrences and lower VA in patients with UG is thought to be related to the low VR-QoL in these patients.

SAP is used as standard to evaluate glaucoma patients. Most visual tasks are performed using binocular vision, and previous studies observed a strong relationship between the binocular VF and the VR-QoL in OAG [35,36]. However, the impact of the VF type (binocular or monocular) on the VR-QoL in UG has not been accurately determined. Intriguingly, we observed a differential impact of the monocular and binocular VF on the VR-QoL in the OAG and UG groups. In the OAG group, the binocular whole IVF field showed the strongest correlation with the composite VFQ-25 score and significant correlations with 11 subscale scores (Figure 2A). This is consistent with previous results from the literature, suggesting that the binocular IVF or the VF of the better eye determines the VR-QoL in OAG [13,14]. In contrast, in UG eyes the inferior VF of the affected eye showed significant correlations with the composite score (r = 0.509, *p* < 0.001) and seven subscale scores (Figure 2B). In glaucoma, the inferior hemifield strongly affects the VR-QoL [12,36]. Cheng et al. [36] showed that the MD of the superior IVF was associated with near activities, whereas the MD of the inferior IVF was associated with general vision, vision-specific role difficulties, and peripheral vision. In this regard, the severe deterioration in the inferior VF seen in the UG patients seems to worsen their VR-QoL compared to those with OAG, as shown in this study (Table 4).

In univariate analyses, age significantly affected the composite VFQ-25 score in the OAG group (Table 3), which is consistent with the previous literature [11,36]. Contrary to the OAG, the average and superior RNFLT were associated with the composite score in the UG group (*p* = 0.014 and 0.010, respectively). In uveitis, the lower VA of the affected eye was one of the main determinants of a lower VR-QoL [17], whereas the better-seeing eye had a greater influence on the VR-QoL in the OAG patients. Previous studies on the VR-QoL of OAG patients suggested that the VF parameters [13,14] and VA of the better seeing eye had the greatest impact on the VR-QoL [15]. It is presumed that, in OAG patients, the better eye compensates for the worse eye in terms of VA and VF loss. However, the compensatory mechanism of the better eye in OAG patients may not be applicable to UG. The exact mechanism underlying this phenomenon needs to be clarified through further studies.

Our study also showed that the average RNFLT was thinner in the OAG group compared to the UG group, whereas the MD of the affected eye showed no significant difference between the OAG and UG groups (Table 1 and Figure 1), implying that UG eyes may show relatively less structural deterioration at a given functional damage. Regarding the less structural deterioration in the UG group, uveitis activity may have influenced the RNFLT measurements. In the patients with hypertensive uveitis, peripapillary RNFL shows dynamic changes over the course of the disease. For example, paradoxical thickening of the RNFL has been associated with active inflammation and an elevated IOP, which is resolved during the quiescent phase and via IOP control [24,37,38]. Therefore, the uveitis-related thickening of the RNFLT in eyes with UG may have led to an overestimation of the RNFLT, resulting in less structural deterioration in these patients. However, in this study, the OCT measurements were performed during the quiescent phase in every patient, and the patients in the UG group all had anterior uveitis without posterior involvement. In addition, Moore et al. [37] reported that, among UG patients, there were no differences in the RNFL measurements between the active and quiescent uveitis subgroups, although there were significant changes in the eyes without glaucoma. The relative functional deterioration in UG eyes may indicate the susceptibility to progressive VF deterioration in these patients. Liu et al. [21] compared the rates of VF loss between the uveitis patients with glaucoma and the patients with POAG and found that the risk of rapid deterioration was twice as high in the former group.

Our findings should be interpreted with consideration of the following limitations. First, due to the inherent limitations of cross-sectional analyses, causality could not be determined. Second, although we measured the RNFLT during the quiescent phase in the UG eyes and excluded uveitic patients with posterior involvement from the UG group, some of the OCT measurements may still have been affected by intraocular inflammation. Third, about 60% of the patients in the OAG group had normal-tension glaucoma, which accords with the very high prevalence (80–90%) of normal-tension glaucoma in northeast Asian countries [39,40]. The patient characteristics may have had a greater impact on hemispheric asymmetry in the OAG group due to the increased susceptibility to glaucoma on the inferior side of the optic nerve head seen in normal-tension glaucoma. Fourth, to better understand the structure–function relationship, we only included patients with recurrent hypertensive anterior uveitis, without posterior involvement, in the UG group. Therefore, the inclusion of posterior uveitis cases in the UG group may lead to different structure–function results. Finally, fifteen patients (21.7%) of the UG patients underwent glaucoma surgery. Although the number is small, it could have affected the VR-QoL in the UG patients.

In conclusion, the eyes with UG had a distinctive structure–function relationship that differentiated them from the OAG eyes. At a given MD, the UG eyes showed less structural deterioration, compared to the OAG eyes. In addition, the patients with UG were associated with a worse VR-QoL than those with OAG, after adjusting for structural and functional metrics. Moreover, while the inferior hemifield of the affected eyes had a major impact on the VR-QoL in the eyes with UG, the binocular IVF was the determining factor of the VR-QoL in the patients with OAG. Our results emphasize the need to pay close attention to the subjective QoL of patients with UG.

## Figures and Tables

**Table 1:** General characteristics and Comparisons of the Ocular Characteristics in eyes with OAG and UG.

	OAG	UG	*p*-Value *
Number of subjects	138	69	
Age (years)	55.16 ± 14.27	52.96 ± 15.80	0.314 *
Gender(M:F)	80:58	47:22	0.175 ^†^
Visual acuity (LogMAR)	0.13 ± 0.18	0.29 ± 0.34	<0.001 *
Average IOP (mmHg)	12.49 ± 3.12	17.25 ± 8.27	<0.001*
Peak IOP (mmHg)	17.8 ± 13.8	34.7 ± 12.7	<0.001*
No. of anti-glaucoma medication (Number)	1.57 ± 0.61	1.67 ± 1.00	0.403 *
Binocular IVF			
Whole IVF (dB)	−3.30 ± 4.36	−2.60 ± 4.87	0.341 *
Superior IVF (dB)	−3.76 ± 5.25	−3.00 ±5.20	0.364 *
Inferior IVF (dB)	−2.83 ± 4.16	−2.21 ± 4.89	0.381 *
Affected eye			
MD (dB)	−9.72 ± 6.33	−10.97 ± 10.32	0.310 *
Superior VF (dB)	−17.49 ± 10.62	−14.11 ± 12.25	0.060 *
Inferior VF (dB)	−12.00 ± 10.62	−12.86 ± 11.81	0.623 *
The other eye MD (dB)	−4.18 ± 4.90	−2.84 ± 4.62	0.075 *
Average RNFLT (μm)	70.54 ± 11.28	75.81 ± 16.58	0.008 *
Superior RNFLT (μm)	75.35 ± 14.82	76.86 ± 17.54	0.518 *
Inferior RNFLT (μm)	70.98 ± 16.93	85.96 ± 18.08	<0.001 *
Average GCIPLT (μm)	63.53 ± 17.18	67.06 ± 15.53	0.154 *

OAG, primary open angle glaucoma; UG, uveitic glaucoma; IOP, intraocular pressure; IVF, integrated visual field; VF, visual field; MD, mean deviation; RNFLT, retinal nerve fiber layer thickness; GCIPLT, ganglion cell inner plexiform layer thickness. * Independent *t*-test, ^†^ Chi-square analyses.

**Table 2:** Comparisons of VFQ-25 score between Patients with OAG and those with UG.

	OAG	UG	*p*-Value *
General health	38.32 ± 20.57	38.95 ± 23.34	0.591
General vision	64.71 ± 15.25	61.40 ± 18.20	0.291
Ocular pain	82.57 ± 16.97	77.91 ± 17.22	0.185
Near vision	81.57 ± 18.78	80.39 ± 20.66	0.330
Distance vision	87.17 ± 14.33	78.29 ± 21.30	0.021
Vision specific social function	94.53 ± 12.39	88.66 ± 18.86	0.021
Vision specific mental health	78.54 ± 17.96	72.14 ± 30.09	0.073
Vision specific role difficulties	81.67 ± 22.07	67.56 ± 25.91	0.001
Vision specific dependency	92.29 ± 17.30	89.09 ± 20.03	0.785
Driving	84.56 ± 22.83	80.90 ± 21.63	0.461
Color vision	95.80 ± 13.06	89.53 ± 17.45	0.001
Peripheral vision	92.70 ± 13.27	84.88 ± 20.51	0.026
Composite score	80.98 ± 11.56	75.52 ± 15.58	0.026

OAG, open angle glaucoma; UG, uveitic glaucoma; VFQ-25, visual function questionnaire-25; OAG, open angle glaucoma; UG, uveitic glaucoma. * General linear model. Age, whole IVF, visual acuity, number of anti-glaucoma medication, VF MD, average RNFLT, and average GCIPT was included in the model to adjust for its potential confounding on the various measurements.

**Table 3:** Univariate Linear Regression Analyses of Factors affecting the Composite Scores in in patients with OAG and those with UG.

	OAG			UG		
	Regression Coefficient	CI	*p* Value	Regression Coefficient	CI	*p* Value
Age	−0.147	−0.283, −0.012	0.033	−0.240	−0.515, 0.034	0.085
Visual Acuity *	−8.820	−19.630, 1.991	0.109	−11.187	−25.862, 3.488	0.131
Average IOP *	0.019	−0.609, 0.648	0.951	−0.027	−0.559, 0.505	0.918
No of medication *	−1.663	−4.850, 1.525	0.304	−5.118	−9.748, −0.487	0.031
Binocular whole IVF	1.287	0.895, 1.680	<0.001	0.778	−1.484, 3.040	0.486
Binocular superior IVF	0.946	0.609, 1.284	<0.001	0.227	−2.016, 2.470	0.837
Binocular inferior IVF	1.324	0.910, 1.738	<0.001	1.091	−0.943, 3.125	0.280
MD *	0.679	0.390, 0.967	<0.001	0.550	−0.029, 1.130	0.062
Superior VF *	0.203	0.022, 0.384	0.029	0.337	−0.192, 0.865	0.202
Inferior VF *	0.226	0.045, 0.408	0.015	0.677	0.216,1.138	0.006
Average RNFLT *	0.160	−0.013, 0.333	0.070	0.377	0.079, 0.676	0.014
Superior RNFLT *	0.071	−0.062, 0.204	0.293	0.392	0.101, 0.683	0.010
Inferior RNFLT *	0.113	−0.002, 0.227	0.054	0.218	−0.066, 0.503	0.129
Average GCIPLT *	0.096	−0.017, 0.209	0.095	0.242	−0.117, 0.600	0.181

OAG, open angle glaucoma; UG, Uveitic glaucoma; IVF, integrated visual field; VF, visual field; IOP, intraocular pressure; MD, mean deviation; RNFLT, retinal nerve fiber layer thickness; GCIPLT, ganglion cell inner plexiform layer thickness; * indicates the unilateral measurement of affected eye.

**Table 4:** Multivariate Linear Regression Analyses of Factors affecting the Composite Scores in OAG and UG.

	Model 1				Model 2			
	Adjusted R^2^	Regression Coefficient	CI	*p* Value	Adjusted R^2^	Regression Coefficient	CI	*p* Value
**OAG**								
Binocular whole IVF	0.238	1.242	0.846, 1.639	<0.001	0.226	1.240	0.825, 1.655	<0.001
Binocular superior IVF	0.188	0.907	0.567, 1.247	<0.001	0.176	0.898	0.541, 1.254	<0.001
Binocular inferior IVF	0.231	1.278	0.862, 1.694	<0.001	0.221	1.259	0.831, 1.688	<0.001
MD *	0.138	0.638	0.345, 0.931	<0.001	0.125	0.641	0.321, 0.962	<0.001
Superior VF *	0.052	0.195	0.016, 0.374	0.033	0.046	0.175	−0.011, 0.361	0.065
Inferior VF *	0.053	0.203	0.021, 0.385	0.029	0.045	0.177	−0.015, 0.369	0.071
**UG**								
MD *	0.135	0.586	0.016, 1.155	0.044	0.116	0.389	−0.386, 1.164	0.310
Inferior VF *	0.272	0.692	0.242,1.242	0.004	0.236	0.596	0.037, 1.156	0.038

OAG, open angle glaucoma; UG, Uveitic glaucoma; IVF, integrated visual field; VF, visual field; MD, mean deviation. Model 1, adjusted for age. Model 2, adjusted for age, visual acuity, number of anti-glaucoma medication. CI, confidence interval; * indicates the unilateral measurement of affected eye.
